# Antinociceptive and Anti-Inflammatory Effects of Total Alkaloid Extract from *Fumaria capreolata*

**DOI:** 10.1155/2015/736895

**Published:** 2015-08-16

**Authors:** Noureddine Bribi, Francesca Algieri, Alba Rodriguez-Nogales, Jose Garrido-Mesa, Teresa Vezza, Fadila Maiza, Maria Pilar Utrilla, Maria Elena Rodriguez-Cabezas, Julio Galvez

**Affiliations:** ^1^CIBER-EHD, Department of Pharmacology, ibs. GRANADA, Center for Biomedical Research (CIBM), University of Granada, Avenida del Conocimiento, S/N, Armilla, 18100 Granada, Spain; ^2^Laboratory of Plant Biotechnology and Ethnobotany, Faculty of Natural Sciences and Life, University of Bejaia, 06000 Bejaia, Algeria

## Abstract

*Fumaria capreolata* is used in traditional medicine in North Africa for its gastrointestinal and anti-inflammatory activities. The present study investigates the effects of total alkaloids extracted from the aerial parts of *Fumaria capreolata *(AFC) on LPS-induced production of proinflammatory mediators (IL-6, IL-1*β*, iNOS, TNF-*α*, COX-2, and MIP-2) in RAW264.7 cells. AFC significantly reduced the inflammatory response inhibiting the production of nitric oxide (NO) and IL-6 in a dose-dependent manner, without affecting the viability of cells, and downregulated mRNA expression of proinflammatory key players: IL-6, IL-1*β*, iNOS, TNF-*α*, and COX-2. AFC antinociceptive and anti-inflammatory properties were also evaluated on the acetic acid- and formalin-induced pain models in mice. AFC oral administration significantly inhibited acetic acid-induced writhes and reduced formalin-induced paw licking time. Therefore, AFC may be a potential candidate for the treatment of inflammatory diseases, such as colitis and arthritis.

## 1. Introduction

Acute inflammation is characterized by heat, redness, pain, and swelling and is a host response to potential harmful agents, including invading pathogens. In the long term, this response can cause progressive tissue damage, which can be the basis of different chronic conditions such as rheumatoid arthritis, asthma, or inflammatory bowel disease. Different mediators have been reported to play a key role in the pathogenesis of these inflammatory disorders, including reactive oxygen and nitrogen metabolites, eicosanoids, or cytokines such as tumor necrosis factor- (TNF-) *α*, interleukin- (IL-) 1*β*, and IL-6, among many others. Therefore, they are important targets for anti-inflammatory molecules [1, 2]. Currently, there are different drugs with reputed efficacy as anti-inflammatory and analgesic; however, and unfortunately, they are not suitable for all patients, particularly those with chronic pain due to limitations of potency, side effects, and lack of tolerability, hence justifying the nonstop search for alternatives [3]. We can find safe anti-inflammatory remedies among medicinal plants, which have been used since ancient times. The beneficial effects of these herbal remedies can be attributed to their content in active compounds with different chemical structure. Among them, alkaloids are one of the largest groups, also with a great chemical diversity. In fact, plants are estimated to produce approximately 12,000 different alkaloids with a wide range of pharmacological properties [4, 5].

The genus* Fumaria* consists of 46 species in the world. Some of them are known by the English terms “fumitory, earth smoke, beggary, fumus, vapor, fumitory, or wax dolls.” Twenty-two of these species are restricted to the Ibero-Mauritanian Region, which includes Algeria, Morocco, and Spain, and many are known to display biological activities [6, 7]. Thus, their use in traditional medicine as antihypertensives and diuretics or to treat different gastrointestinal disorders, including constipation or functional diseases of the biliary system, has been reported. These plants have been characterized by the presence of various types of isoquinoline alkaloids in their composition, particularly aporphine, protoberberine, protopine, and benzophenanthridine [8]. In a previous study, it was reported that the total alkaloid fraction from* Fumaria capreolata* exerted antioxidant activity, without any toxicity in vivo, since doses up to 2,000 mg/kg did not cause any acute adverse effect when administered orally to mice [9]. The aim of this study was to further characterize the biological activities of the alkaloid fraction from* Fumaria capreolata*, by evaluating its anti-inflammatory properties both in vitro and in vivo.

## 2. Materials and Methods

### 2.1. Drugs and Chemicals

All substances were purchased from Sigma-Aldrich Chemical (St. Louis, MO, USA), unless otherwise stated. The test substances were dissolved in distilled water and prepared fresh daily for administration to the animals.

### 2.2. Extraction of Alkaloids

Aerial parts of* Fumaria capreolata* were collected from Bejaia area, in the northeast of Algeria in May 2013 when they were at the flowering and fruit setting stage. The plant was authenticated by Professor F. Benabdesselam (Laboratory of Plant Biotechnology and Ethnobotany, University of Bejaia, Algeria) and voucher specimen was deposited (reference number: FC015). The alkaloid extract of* Fumaria capreolata* (AFC) was obtained following the procedure previously reported [10]. Briefly, the aerial parts of the plant were dried in an oven at 40°C overnight and grounded into fine powder using a grinder. The powder samples (1 kg) were extracted with ethanol in a Soxhlet apparatus for 8 h, evaporated under reduced pressure, acidified with 2.5% HCL to pH 1-2 and filtered, and then stored overnight at room temperature. The aqueous acid solution was adjusted to pH = 9.5 with concentrated ammonium hydroxide and extracted with dichloromethane. The extracts were dried over magnesium sulphate and the solvent evaporated to get a crude extract of total alkaloids. After evaporation the yield of each fraction was calculated, and the alkaloid extract of* Fumaria capreolata* (AFC) obtained was stored at 4°C until use.

## 3. Antinociceptive Effects of AFC

### 3.1. Animals

Female albino NMRI mice (22–28 g) were purchased from the Laboratory Animal Service of the University of Constantine (Algeria) and housed under standard laboratory conditions with free access to tap water and food. All behavioral tests were conducted during the light cycle and all procedures used in the present study were carried out in accordance with the current guidelines for the care of laboratory animals and the ethical guidelines for investigations of experimental inflammation and pain in conscious animals [11], following the directive number 2010/63/EU of 22 September 2010.

### 3.2. Acetic Acid-Induced Writhing Response

The writhing test was carried out as previously described [12]. Mice were randomly assigned to five different groups (*n* = 6), and after an overnight fasting period, they were pretreated with AFC (100, 250, or 500 mg/kg, p.o.), diclofenac (100 mg/kg, p.o.), or distilled water (control group, p.o.) 60 min before the acetic acid injection (10 mL/kg body weight, i.p.). Immediately after the injection of acetic acid, each animal was placed in a transparent plastic observation chamber. Five minutes after the administration of the acetic acid, the number of writhes and stretching movements (contraction of the abdominal musculature and extension of hind limbs) of each mouse was counted for a period of 15 min. The percentage of inhibition of writhing was calculated and compared with the control group using the expression: inhibition (%) = (WC − WT)/WC × 100; WC: mean of writhing (control); WT: mean of writhing (test).

### 3.3. Formalin-Induced Paw Licking

The formalin test was performed as previously reported [13, 14]. Briefly, overnight fasted mice were divided into five groups (*n* = 5), which received distilled water (10 mL/kg, p.o.), AFC (100, 250, and 500 mg/kg, p.o.), or acetaminophen (100 mg/kg, p.o.) 1 h before formalin injection (20 *μ*L of 1% formalin) under the plantar surface of the right hind paw. The mice were then placed in a transparent box for observation, and the time spent licking the injected paw was measured and considered as an indication of inflammatory-associated pain. The first phase of the nociceptive response normally peaks 0–5 min after injection and the second phase 15–30 min after.

## 4. Anti-Inflammatory Effects of AFC

### 4.1. Cell Culture and Determination of Cell Viability

RAW 264.7 cells (murine macrophages) were obtained from the Cell Culture Unit of the University of Granada (Granada, Spain) and cultured in Dulbecco's Modified Eagle Medium (DMEM), supplemented with 10% FBS and 2 mM l-glutamine, in a humidified 5% CO_2_ atmosphere at 37°C. Cytotoxicity evaluation of the AFC was carried out in 96-well plates using the MTT assay [15]. RAW 264.7 cells were incubated in the presence of different concentrations (12.5, 25, 50, and 100 *μ*g/mL) of AFC and 100 ng/mL of LPS for 18 h. The MTT solution was then added to each well and incubated for another 1 h, when the absorbance of the solution at 490 nm was measured. The cellular viability was determined from the absorbance value and compared with that of the untreated control cells.

### 4.2. Determination of NO and IL-6 Production

RAW 264.7 cells were cultured with increasing concentrations of AFC (12.5, 25, 50, and 100 *μ*g/mL) and LPS (100 ng/mL). After 24 hours, the supernatants were collected. The nitrite accumulation in the supernatant was determined by the Griess assay [16]. Briefly, equal volume of cell-free culture media (100 *μ*L) was reacted with Griess reagent (100 *μ*L), and the absorbance at 540 nm was measured. The level of IL-6 in cell culture media was determined as well using commercial available ELISA kits (R&D) according to the manufacturer's instructions.

### 4.3. Analysis of Gene Expression by RT-PCR

RAW 264.7 cells were cultured with increasing concentrations of AFC (12.5, 25, 50, and 100 *μ*g/mL) for 2 hours prior to LPS (100 ng/mL) stimulation. Three hours later, cells were collected and total RNA extracted using Trizol (Ambion, Austin, TX, USA), following the manufacturer's instructions. RNA samples were quantified with the NanoDrop 2000 Spectrophotometer (Thermo Scientific, Wilmington, DE, USA), and 2 *μ*g of RNA was reverse transcribed using oligo (dT) primers (Promega, Madison, WI, USA). Real-time quantitative PCR amplification and detection were performed on optical-grade 48-well plates in an Eco Real-Time PCR System (Illumina, CA, USA) with 20 ng of cDNA, the KAPA SYBR FAST qPCR Master Mix (KapaBiosystems, Inc., Wilmington, MA, USA), and specific primers at their annealing temperature (Table 1). To normalize mRNA expression, the expression of the housekeeping gene glyceraldehydes 3-phosphate dehydrogenase (GAPDH) was measured for comparative reference. The mRNA relative quantitation was calculated using the ΔΔCt method.

### 4.4. Statistical Analysis

All data were expressed as mean ± standard error of the mean (SEM). The statistical analysis of all the observations was carried out using one-way ANOVA followed by multiple comparison test of* Dunnett's*, where necessary. A difference of *p* < 0.05 was considered statistically significant compared with the negative control (untreated).

## 5. Results and Discussion

Nonsteroidal anti-inflammatory drugs (NSAIDs) are the most widely prescribed medications for the management of painful conditions associated with inflammation, but they can frequently cause gastrointestinal damage that can even lead to ulcers and hemorrhage [17]. As an alternative, plant based medicines are more and more used, based on their reputed efficacy and supposed fewer adverse effects [18]. In fact, medicinal plant extracts are composed of different constituents that may exert complementary properties and mechanisms of action, thus providing synergistic effects. However, they may also result in an increased risk of harmful effects [19]. In this context, the use of more purified fractions, obtained from plant extracts that have been long used in traditional medicine, may be of special interest. In the present study, the potential anti-inflammatory effects of an alkaloid fraction from* Fumaria capreolata* (AFC) have been evaluated both in vitro and in vivo. With this aim, firstly we have tested AFC on LPS-stimulated macrophage activity, by using the RAW 264.7 cell line, and secondly, we have assayed the analgesic properties of AFC by using two different in vivo experimental models: acetic acid-induced writhing test and formalin-induced licking test in mice. The results obtained in the present study revealed that total alkaloid extract of aerial parts of* Fumaria capreolata* possessed anti-inflammatory and antinociceptive effects.

**Figure 1:**
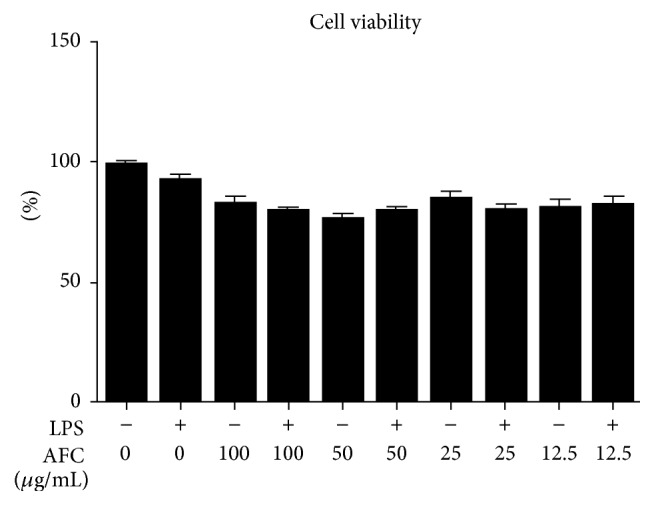
Effects of AFC on cell viability in RAW 264.7 cells treated with LPS (100 ng/mL) for 24 h. Data are expressed as means ± SEM (*n* = 6).

Of note, in a previous study, this alkaloid fraction was reported to show a very low toxicity when administered orally to mice [9]. The in vitro experiments performed in the present study confirmed these observations. Thus, the incubation of RAW 264.7 cells with increasing concentrations of AFC for 24 h revealed that the cell survival rate was greater than 75% at all concentrations assayed, and no significant differences were noted when compared with LPS treated cells (Figure 1).

**Figure 2:**
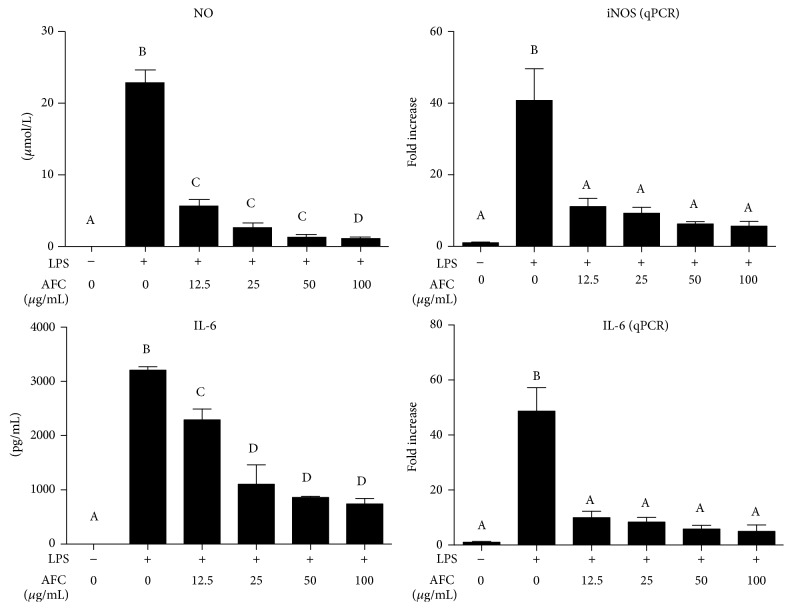
Effects of AFC on NO production (Griess reaction), iNOS expression (qPCR), IL-6 production (ELISA), and IL-6 expression (qPCR) in RAW 264.7 cells treated with LPS (100 ng/mL). Data are expressed as means ± SEM (*n* = 6). Columns with different letter statistically differ (*p* < 0.05).

**Figure 3:**
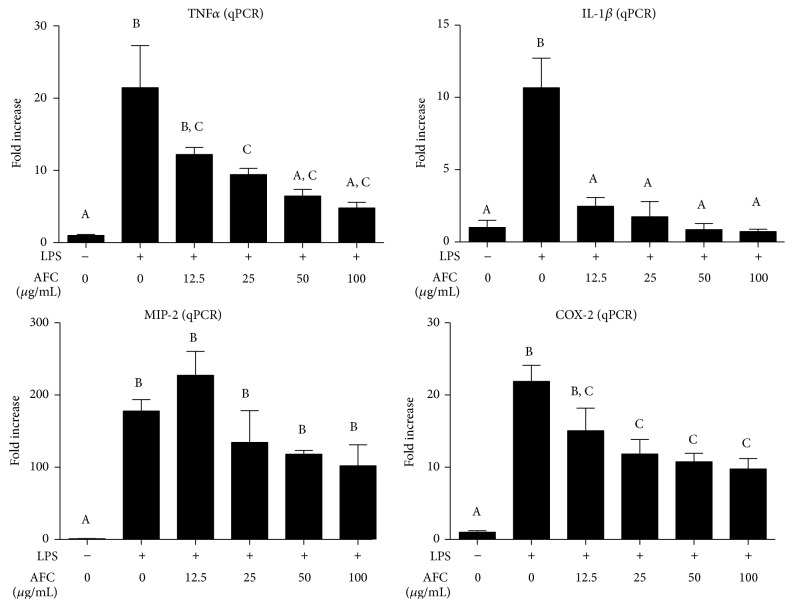
Effects of AFC on TNF-*α*, IL-1*β*, MIP-2, and COX-2 expressions determined by qPCR in RAW 264.7 cells treated with LPS (100 ng/mL). Data are expressed as means ± SEM (*n* = 6). Columns with different letter statistically differ (*p* < 0.05).

Macrophages play a key role in maintaining the homeostasis of the organism and they are activated during inflammation and pathogen challenge. They experience some biochemical and morphological modifications that qualify them to perform their professional functions [20]. On the other hand, LPS is an outer membrane component of Gram-negative bacteria and a potent activator of monocytes and macrophages, through its binding to surface toll-like receptor 4 (TLR4), triggering the overexpression and subsequent secretion of a variety of inflammatory products, including TNF-*α*, IL-1*β*, and IL-6, as well as high amounts of nitric oxide (NO), which contribute to the pathophysiology of inflammatory conditions [21]. When NO production was evaluated in LPS-stimulated RAW 264.7 cells, AFC was able to significantly decrease it in a concentration dependent manner (Figure 2). Since LPS induces the expression of inducible nitric oxide synthase (iNOS) in macrophages and the synthesis of iNOS protein correlates with NO production [22], the impact of AFC on iNOS expression was also evaluated in these LPS-stimulated cells. The results revealed that this alkaloid extract significantly downregulated the increased expression of this inducible enzyme (Figure 2). Similarly, AFC inhibited dose-dependently both the production and the expression of the proinflammatory cytokine IL-6 in these macrophage cells after incubation with LPS (Figure 2). Moreover, when the mRNA expressions of other proinflammatory markers were evaluated in these cells, AFC also reduced the levels of IL-1*β*, TNF-*α*, and cyclooxygenase- (COX-) 2 dose-dependently (Figure 3). Considering all the above, this in vitro assay may resemble a Gram-negative bacteria-mediated inflammation in macrophages, and the results obtained demonstrate that AFC was able to significantly reverse the induction of the expression of all the markers evaluated, including inducible enzymes (iNOS and COX-2) and proinflammatory cytokines (IL-1*β*, TNF-*α*, and IL-6). This mechanism could justify the anti-inflammatory effect for this alkaloid fraction, since the macrophages have a key role in different inflammatory conditions [23].

**Figure 4:**
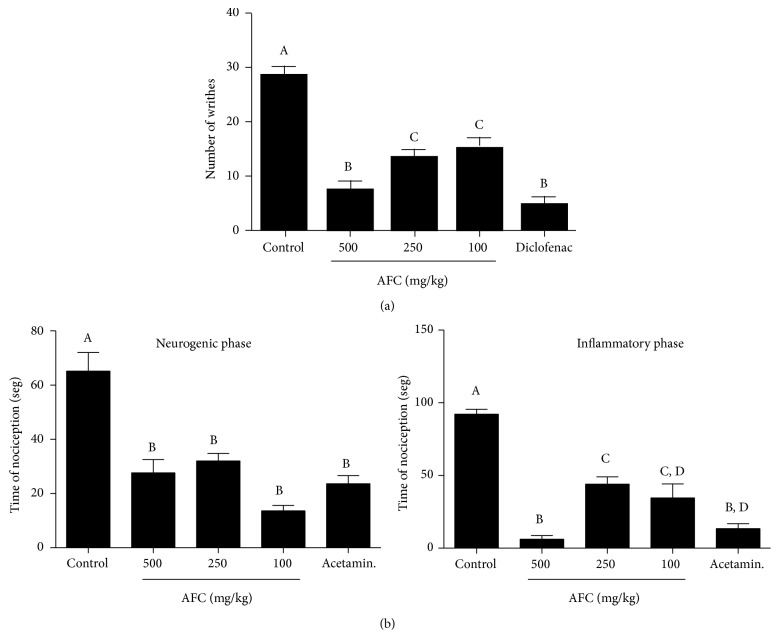
Effects of oral administration of AFC on (a) abdominal writhes induced by acetic acid in mice and (b) neurogenic phase and inflammatory phase on formalin-induced pain in mice. Data are expressed as means ± SEM (*n* = 6). Columns with different letter statistically differ (*p* < 0.05).

The writhing biting and licking response to acute nociception has been used to test the antinociceptive activity of AFC in mice. In the writhing test, intraperitoneal injection of 1% acetic acid evidently resulted in writhing reflex in untreated control mice. The alkaloid extract of* Fumaria capreolata* produced a significant inhibition of the writhing reaction induced by acetic acid when compared to the control group, this effect being dose-dependent, and with a similar efficacy at the highest dose of AFC assayed (500 mg/kg) to that obtained with diclofenac (100 mg/kg) (Figure 4(a)). It has been reported that the injection of acetic acid into the peritoneal cavity promotes an increase of cyclooxygenase and lipoxygenase products in peritoneal fluids as well as the release of many other inflammatory mediators including bradykinin and substance P but also proinflammatory cytokines like TNF-*α*, IL-1*β*, and IL-8 which finally stimulate the primary afferent nociceptors entering the dorsal horn of the central nervous system. Acetic acid induces inflammatory pain by causing capillary permeability and liberating endogenous substances that stimulate pain nerve endings [24, 25]. The antinociceptive activity of AFC in inflammatory conditions was confirmed after its administration to mice submitted to formalin-induced paw edema. It has been well described that, during the first phase, the injection of formalin into the subplantar tissue of the hind paw of mice produces a neurogenic nociceptive response of biting and licking of the damaged paw, which lasted in the untreated control group more than one minute. The administration of different doses of the alkaloid extract resulted in a significant reduction of the biting and licking time, thus revealing an antinociceptive potential against formalin-induced pain; although no dose-response relationship was achieved, the efficacy obtained was similar to that observed with acetaminophen, the analgesic used as positive control (Figure 4(b)). Of note, the beneficial effect exerted by AFC was maintained during the second phase of nociception that characterizes this experimental model (Figure 4(b)), which is associated with the release of inflammatory mediators that clearly contributes to the pain [14–26]. Total alkaloids of* Fumaria capreolata* possess antinociceptive activity against chemically induced nociception associated with inflammation. Among the different alkaloids present in the extract, protopine, an isoquinoline alkaloid, is the most medicinally active phytochemical, as it is reported to possess numerous pharmacological actions [27].

## 6. Conclusion

In conclusion, the present study suggests that total alkaloid fraction from* Fumaria capreolata* presents anti-inflammatory effects since it inhibits the expression and/or release of different anti-inflammatory mediators when evaluated in vitro in LPS-stimulated macrophage RAW 264.7 cells, which could contribute to its potential analgesic effect. This was supported by the results obtained in vivo, which showed that AFC significantly exerted analgesic effects in two experimental nociceptive models in which the release and/or actions of different inflammatory mediators, like vasoactive substances (histamine, serotonin, and kinins) as well as prostaglandins, play a key role.

## Figures and Tables

**Table 1:** Primer sequences used in RT-PCR assays in RAW264.7 cells.

Gene	Primers sequences	Annealing *T* (°C)
*IL-6*	FW 5′-CTTCCCTACTTCACAAGTC-3′	60
	RV 5′-CTCCATTAGGAGAGCATTG-3′	

*TNF-α*	FW 5′-AACTAGTGGTGCCAGCCGAT-3′	56
	RV 5′-CTTCACAGAGCAATGACTCC-3′	

*iNOS*	FW 5′-GTTGAAGACTGAGACTCTGG-3′	56
	RV 5′-GACTAGGCTACTCCGTGGA-3′	

*IL-1β*	FW 5′-TGATGAGAATGACCTCTTCT-3′	55
	RV 5′-CTTCTTCAAAGATGAAGGAAA-3′	

*COX-2*	FW 5′-GGGTTGCTGGGGGAAGAAATG-3′	67
	RV 5′-GGTGGCTGTTTTGGTAGGCTG-3′	

*MIP-2*	FW 5′-CAGTTAGCCTTGCCTTTGTTCAG-3′	62
	RV 5′-CAGTGAGCTGCGCTGTCCAATG-3′	

*GADPH*	FW 5′-CCATCACCATCTTCCAGGAG-3′	60
	RV 5′-CCTGCTTCACCACCTTCTTG-3′	
